# Supplementary material for: The causal relationship between the human gut microbiota and pyogenic arthritis: a Mendelian randomization study
Source: Front Cell Infect Microbiol. 2024 Nov 26;14:1452480. doi: 10.3389/fcimb.2024.1452480 (PMC11629706; doi:10.3389/fcimb.2024.1452480)
Supplement: Supplementary file 1 [file DataSheet1.zip › mendelian test/ebi-a-GCST90027774.csv_leave_one_out.pdf]

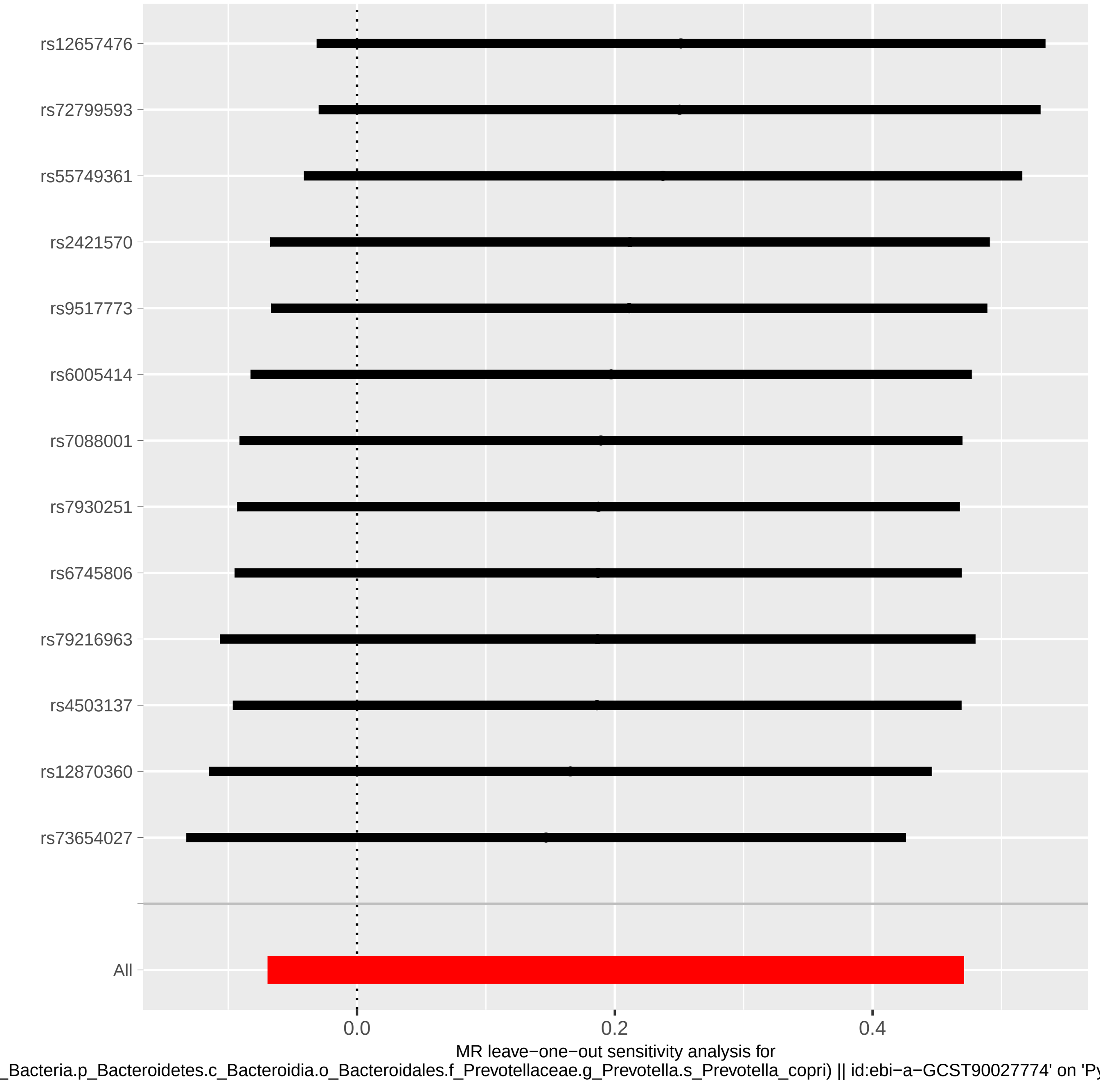

rs12657476
rs72799593
rs55749361
rs2421570
rs9517773
rs6005414
rs7088001
rs7930251
rs6745806
rs79216963
rs4503137
rs12870360
rs73654027
All
0.0
0.2
0.4
MR leave−one−out sensitivity analysis for
_Bacteria.p_Bacteroidetes.c_Bacteroidia.o_Bacteroidales.f_Prevotellaceae.g_Prevotella.s_Prevotella_copri) || id:ebi−a−GCST90027774' on 'Py
